# Nanoscale Heat Flow and Thermometry in Laser-Heated Resonant Silicon Mie Nanospheres Probed with Spatially Resolved Cathodoluminescence Spectroscopy

**DOI:** 10.1021/acsphotonics.5c01417

**Published:** 2025-09-20

**Authors:** Saskia Fiedler, Loriane Monin, Hiroshi Sugimoto, Minoru Fujii, Wiebke Albrecht, Albert Polman

**Affiliations:** † Department of Sustainable Energy Materials, 55952 NWO-Institute AMOLF, Science Park 104, 1098 XG Amsterdam, The Netherlands; ‡ Department of Electrical and Electronic Engineering, Graduate School of Engineering, 12885 Kobe University, Rokkodai, Nada, Kobe 657-8501, Japan

**Keywords:** electron microscopy, cathodoluminescence, nanothermometry, Mie resonances, heat flow simulations

## Abstract

Many nanoscale technologies depend critically on precise knowledge and control of local temperature and heat flow, making robust nanothermometry essential for designing, optimizing, and ensuring the reliability of next-generation devices. In this work, we introduce a correlative method that combines laser excitation with scanning electron microscopy-based cathodoluminescence (SEM-CL) to probe photothermal effects *in situ* with nanoscale spatial resolution. We analyze the spatially resolved CL (30 keV) of resonant Mie modes in single silicon nanoparticles under continuous-wave laser irradiation (λ = 442 nm). The 235–250-nm-diameter crystalline nanospheres, placed on a Si_3_N_4_ membrane, show a strong electric quadrupole CL resonance of which the peak wavelength reversibly red-shifts upon laser-induced heating. A temperature of up to 585 ± 12 °C is derived from the spectral shifts for the highest laser power used (9.6 mW, ∼1 × 10^6^ W/cm^2^ at the substrate). Numerical heat flow simulations show that the measured steady-state temperatures are consistent with a geometry in which heat flow occurs through a contact area of up to 100 nm^2^, depending on laser power, between the Si nanosphere and the Si_3_N_4_ membrane. We postulate that this contact forms by reshaping of the particle–membrane geometry as it heats up in the initial phase of the laser irradiation, leading to an equilibrium geometry that results in the measured steady-state temperature. This work shows that CL of resonant nanostructures in combination with simulations can serve as sensitive probes of temperature and thermal conductivity. Spatially resolved CL nanothermometry in a SEM enables studies of nanoscale thermal properties of a wide range of device geometries such as electronic integrated circuits, surface catalysts, photovoltaic devices, and more.

## Graphical abstract

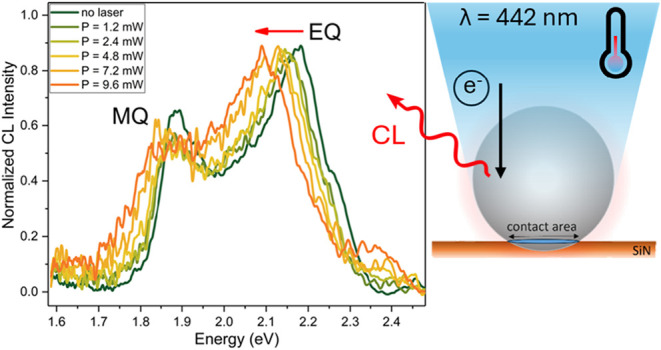

## Introduction

Many processes that are driven by phenomena that occur at the nanoscale are sensitively dependent on the distribution of temperature and heat flow at the nanoscale. For example, the operation of electronic integrated circuits is sensitive to thermal hotspots created by a dense current flow. The efficiency of photothermal chemical reactions is critically dependent on heat flow between the catalyst and the substrate.^1^ And the lifetime of photovoltaic devices is crucially dependent on effective heat flow across nanostructured surface coatings and interfaces. However, measuring temperature at the nanoscale is challenging, as conventional bulk temperature probes cannot be integrated well with the nanoscale geometries. Meanwhile, as devices continue to shrink in size, the demand for precise, local measurements of temperature and heat flow that can be used to design and optimize these devices is growing.

In recent years, several efforts have been made to probe heat transport and temperature at the nanoscale.^2^ For example, noncontact optical techniques, such as thermoreflectance^3,4^ and Raman spectroscopy,^5^ have demonstrated high sensitivity; however, their spatial precision is limited by the optical diffraction limit. High-resolution local probe techniques such as near-field scanning optical microscopy^6^ and scanning thermal microscopy^7–9^ have enabled local temperature mapping with precision down to tens of nanometers. However, these techniques lack depth resolution and suffer from the undesired influence of the probe itself.

Transmission electron microscopy (TEM) has been utilized for nanothermometry by using electron diffraction to probe the temperature from lattice strain.^10^ Electron energy loss and gain spectroscopy (EELS/EEGS) in the TEM has been used to derive the temperature from the Boltzmann distribution over phonon bands^11–14^ and spectral shift of Mie resonances in high-index nanostructures.^15^ Another EELS-based technique is plasmon energy expansion thermometry,^16–18^ which utilizes the spectral position of the bulk plasmon to determine the temperature, as demonstrated for various materials, including silicon nanoparticles (NPs).^17^ Similarly, TEM-based cathodoluminescence (CL) spectroscopy has been employed to measure temperature ratiometrically.^19,20^ The TEM-based techniques offer high spatial resolution in the measurement of thermal properties due to the nanoscale size of the electron probe. Yet, they require special preparation methods to create electron-transparent sample lamellae, which is sometimes undesired.

Recently, time-modulated CL spectroscopy in the scanning electron microscope (SEM) has been used to probe temperature and thermal conductivity in semiconductors from thermal bandgap shifts^21^ as well as thermal broadening of the optical signature of color centers in nano diamonds.^22^ The advantage of using SEM is that sample preparation is often relatively simple and can be performed on devices under operation. Furthermore, depth-resolved information can be achieved by varying the electron energy.^23^ So far, these techniques have not enabled *in situ* measurements under illumination with light, which is essential in photothermal studies, for example.

In this paper, we introduce the use of SEM-CL in combination with laser excitation in the electron microscope. This allows for *in situ* investigation of photothermal effects with nanoscale spatial resolution, while concurrently probing optical properties through CL spectroscopy. Using this approach, we demonstrate the measurement of thermal properties at the nanoscale by using resonant optical phenomena probed by SEM-CL.

We build on previous work in which a 10–30 keV electron beam was used to excite electric and magnetic multipole Mie resonances in individual crystalline Si nanoparticles (NPs),^24–27^ which exhibit low nonradiative losses and hence relatively narrow spectral line widths.^28,29^ Earlier work has shown that the resonant Mie modes of Si NPs undergo a spectral shift under heating due to the temperature dependence of the optical constants of Si.^15^ Here, we perform SEM-CL spectroscopy on Si NPs on a Si_3_N_4_ membrane that are heated with a continuous-wave (cw) laser and use the spectral resonance shifts to probe thermal properties.

To enhance the sensitivity of our measurements, we perform spatially selective CL studies, where a 30 keV electron beam selectively excites the spectrally narrow electric quadrupole (EQ) mode. We use nanoscale heat flow simulations and compare these to temperature measurements from the spectral CL shifts. This enables us to precisely identify the nanoscale thermal contact area between each Si NP and the membrane. We find temperature increases up to 585 ± 12 °C and observe that the contact area varies strongly between Si NPs. This study demonstrates how SEM-CL of resonant nanostructures is a powerful method to probe local thermal properties. The technique can be used on a broad range of resonant nanostructures, such as dielectric waveguides in integrated optics, plasmonic nanoparticles for catalysis and photovoltaics, and metal nanowires in electronic integrated circuits.

## Experimental Methods and Simulations

We drop-cast Si NPs with diameters ranging from 100 to 300 nm onto a 15-nm-thin Si_3_N_4_ membrane (from TedPella). The synthesis procedure for the crystalline Si nanospheres can be found elsewhere.^30^ Figure 1 shows the schematic of the SEM-CL system used for the CL collection and light in-coupling into the SEM chamber. The CL measurements were performed in an FEI Quanta650 SEM (Thermo Fisher) equipped with a Delmic Sparc CL system, which consists of a parabolic mirror to collect the emitted light from the sample and a spectrometer with a Andor Newton CCD camera for spectral analysis. We used an acceleration voltage of 30 keV and an electron beam current of 4 nA in all measurements. CL maps were recorded with a pixel size of 10 nm at an exposure time of 100 ms each.

**Figure 1:**
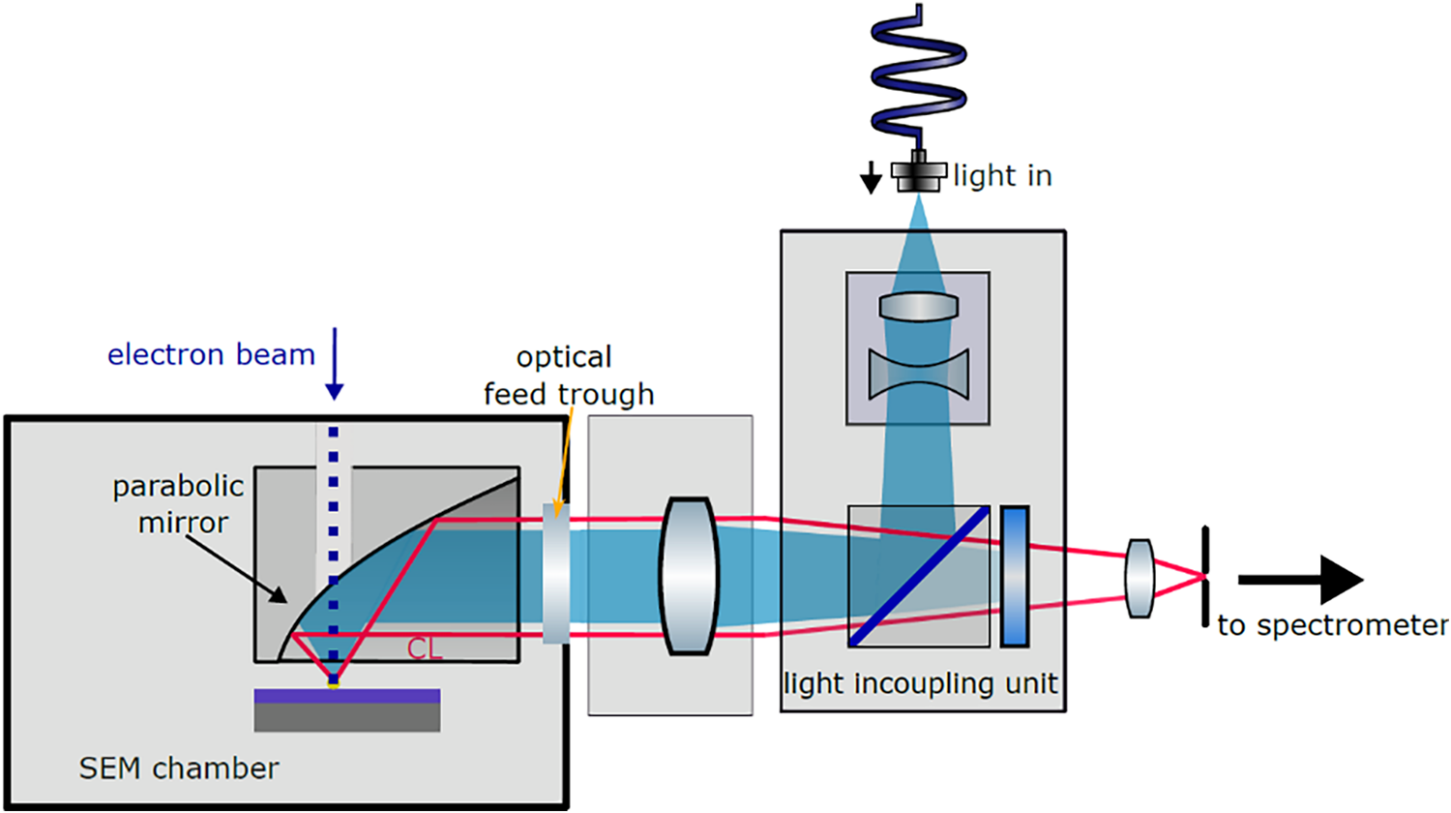
Schematic of light in-coupling into the SEM and the CL collection.

Light from a single-mode-fiber-coupled laser (λ = 442 nm, PicoQuant Taiko) was injected into the SEM chamber using a precollimation lens pair and a dichroic beam splitter (Semrock). We used the parabolic CL collection mirror to focus the laser beam onto the sample (focal diameter ∼1 μm) centered around the electron beam position. The laser output power was measured with a fiber-coupled power meter (Thorlabs), and we estimate a 20% loss in power by the in-coupling optics. The CL emission was filtered using notch (442 nm, Semrock) and long-pass filters (>500 nm, Thorlabs) in the collection path, and CL spectra were corrected for remaining background signals.

Numerical heat flow simulations were performed with the COMSOL program v5.5. The simulated geometry consisted of a 250-nm-diameter silicon nanosphere placed on a 15-nm-thin Si_3_N_4_ membrane suspended in vacuum. To ensure convergence, the size of the simulation domain was set to 10 by 10 μm^2^; details are described in the Supporting Information (SI; Figures S11–S14 and Table S1). The heat generation in the NP was calculated using the NP absorption cross section simulated with MNPBEM17.^31^ Calculations show that in steady state, there is negligible spatial dependence on the temperature inside the Si NP, due to the small NP size and high thermal conductivity of Si.

## Results and Discussion

We disperse Si NPs with a diameter in the range 230–250 nm on a 15-nm-thin Si_3_N_4_ membrane. NPs in this size range exhibit strong magnetic and electric dipole (MD, ED) and quadrupole (MQ, EQ) modes in the visible and near-infrared spectral range. We use the quadrupole modes in our temperature studies as their narrower line widths enable the most sensitive measurements.

Figure 2a shows experimental CL data averaged over the rim of the Si NP at electron impact parameter *b* = 95 nm for a Si NP with a diameter of 235 ± 2 nm excited at 30 keV; the secondary electron (SE) image of the Si NP is depicted in Figure 2b. The corresponding calculated CL spectrum, allowing for the decomposition and thereby identification of the different Mie modes is also shown in Figure 2a.^24,26,32^ The spectra exhibit two sharp spectral features which can be assigned to the MQ and EQ modes, as well as a broad low-energy shoulder associated with the ED mode. We assign the discrepancies in peak wavelength and line width between theoretical and experimental spectra to imperfections in roundness, and potential higher optical losses due to the polycrystalline structure of the Si NPs, which would modify the optical constants compared to those used in the simulations. Simulations show that coupling to the substrate has a negligible effect on the resonances (cf. Figure S8, private communication with P. Elli Stamatopoulou).

**Figure 2:**
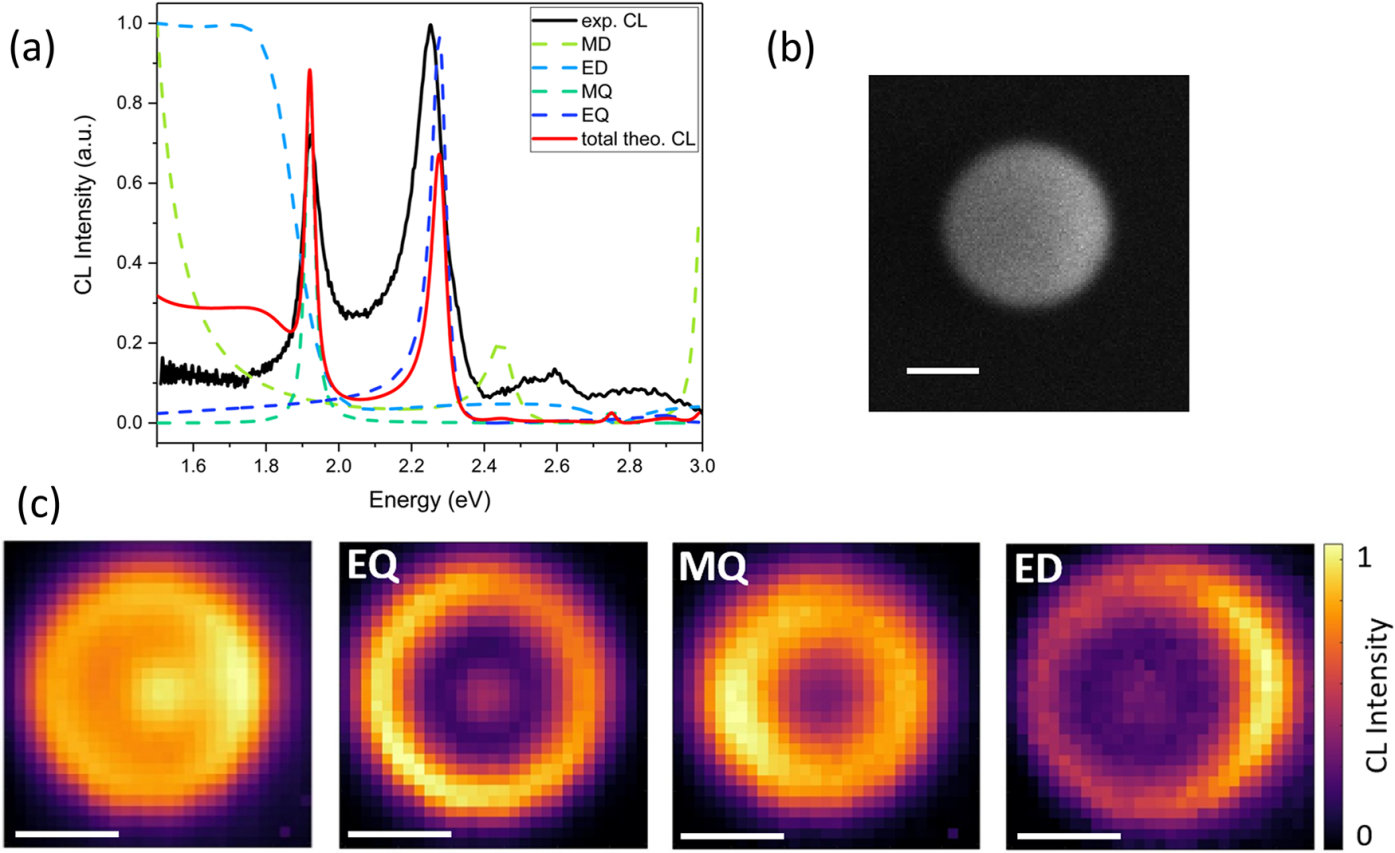
SEM-CL measurements and simulations for a Si nanosphere (*d* = 235 ± 2 nm) on a 15-nm-thin Si_3_N_4_ membrane at 30 keV. (a) Experimental CL spectrum (black) averaged over the rim of the nanosphere, at an impact parameter *b* = 95 nm. Corresponding CL spectrum (red) with *b* = 95 nm calculated using the electron-mode coupling model with individual modal contributions indicated (dashed blue lines).^26^ (b) Secondary electron image. (c) Experimental CL maps, from left to right: spectrally integrated CL map (1.4–3.0 eV), electric quadrupole (EQ) mode at 2.25 ± 0.01 eV, magnetic quadrupole (MQ) mode at 1.92 ± 0.01 eV, and electric dipole (ED) mode at 1.80 ± 0.02 eV. CL intensity is normalized to the maximum for each CL map separately. All scale bars denote 100 nm.

Figure 2c displays CL maps of the Si NP. The spectrally integrated map exhibits a strong CL intensity variation over the NP area. To distinguish the contributions from the individual Mie modes, we also plot CL maps over the spectral ranges of the EQ (2.25 ± 0.01 eV), MQ (1.92 ± 0.01 eV), and ED (1.80 ± 0.02 eV), each revealing a distinct spatial CL excitation distribution for each mode.

Next, we couple laser light into the SEM chamber and repeat the CL experiments with a similarly sized Si NP (*d* = 250 ± 2 nm) at laser output powers increasing from 1.2 to 9.6 mW, resulting in stepwise increased temperatures of the uniformly heated Si NP. For the highest laser power, the estimated power density at the substrate is 1 × 10^6^ W/cm^2^. The normalized CL spectra integrated over the rim of the NP (*b* = 90–120 nm) are presented in Figure 3a. In the analysis, a reduced spectral collection window (1.6–2.5 eV) was chosen to avoid the collection of undesired background emission introduced by the laser. The CL spectrum without laser illumination (dark green) shows MQ and EQ resonances centered at 1.87 and 2.17 eV, respectively. In our further analysis, we spatially select the CL emission from the outer ring of the NP to benefit from the enhanced relative contribution of the EQ signal. Figure 3a clearly shows an increasing spectral red shift of the EQ resonance as the laser power is increased from 1.2 to 9.6 mW. A spectral broadening of the EQ mode is also observed. We note that the laser-induced background spectra and the corresponding spectral correction procedure to retrieve the CL spectra in Figure 3a are described in the SI (cf. Figures S4–S6).

**Figure 3:**
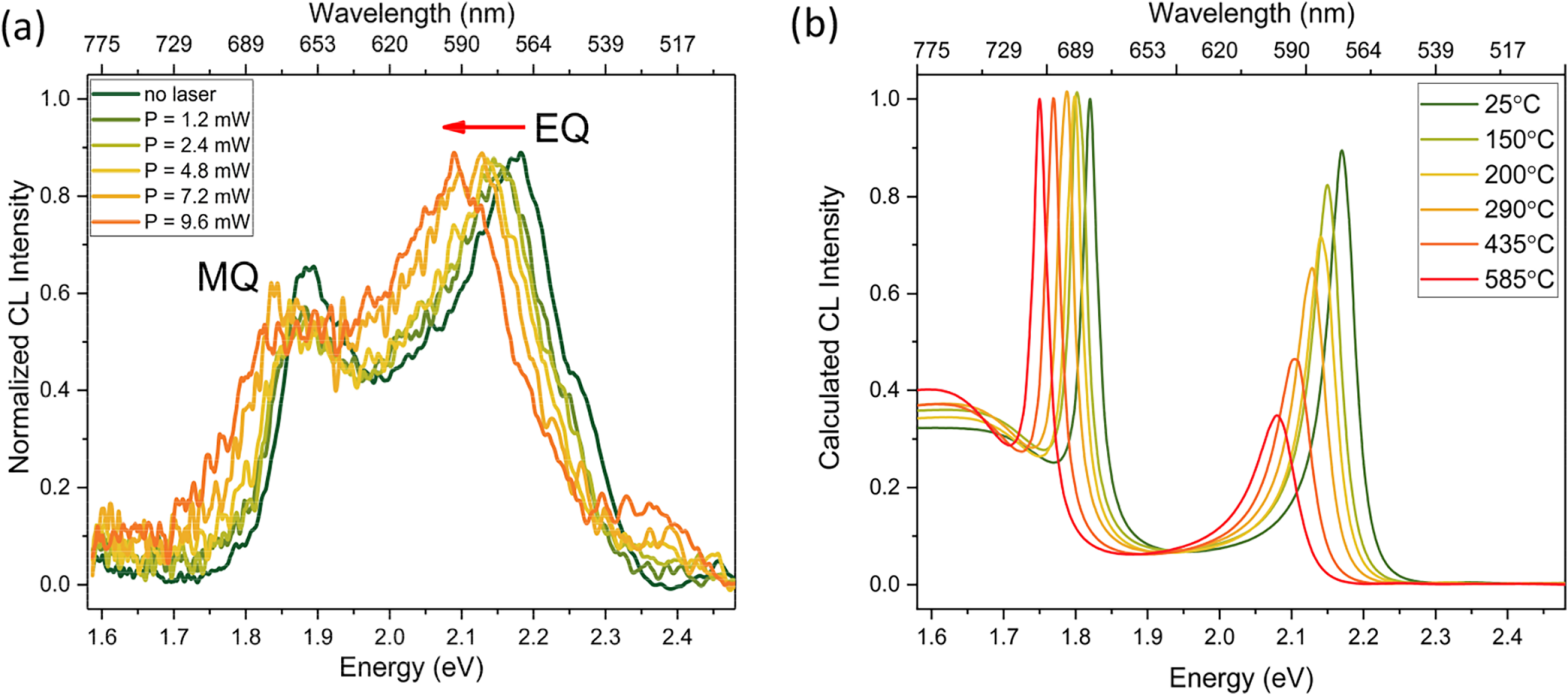
(a) Normalized CL spectra (30 keV) of an individual Si NP (*d* = 250 ± 2 nm) on a 15-nm-thin Si_3_N_4_ membrane averaged over the rim (*b* = 90–120 nm) of the NP, exposed to continuous-wave laser light (λ = 442 nm) at powers in the range 0–9.6 mW (spot size 1 μm). MQ and EQ resonance undergo a spectral shift with increasing laser output power, indicating heating of the Si NP. (b) CL spectra for the same impact parameter range, calculated using Mie theory with varying refractive index according to the shown temperatures.

Figure 3b displays the calculated CL spectra of a 250-nm-sized Si NP in vacuum at temperatures ranging from room temperature to 585 °C using the model from Jellison et al.^33^ for the temperature-dependent refractive index of silicon (Figure S3). The calculations show that in this temperature range the resonant Mie modes experience a clear shift in peak energy and broadening in line width due to the increase in the real and imaginary part of the refractive index of Si, respectively.

To further analyze the temperature-induced spectral changes, we apply a Lorentzian fit to the experimental EQ spectra to determine both the peak energy and the full width at half-maximum (FWHM) of the EQ modal spectrum measured at each laser output power. We observe a red shift of the EQ from 2.17 eV without the laser to 2.08 eV at 9.6 mW, and a peak broadening from 198 to 257 meV FWHM. The extracted peak positions and line widths, along with their fitting uncertainties, are presented as a function of laser power in Figure S7 of the SI, showing a linear relationship. Next, we calculate the EQ peak energies and line widths for the full temperature range from room temperature to the melting point of silicon, using the same model as in Figure 3b. We then assign a temperature to each experimentally derived EQ mode from the spectra in Figure 3a by matching the measured spectral shifts to those predicted by the temperature-dependent Mie model. Figure 4 shows the relation between EQ peak energy and temperature derived using this method, allowing us to assign a temperature of 585 ± 12 °C to the Si NP at the highest laser output power of 9.6 mW. The uncertainty associated with the assigned temperature reflects both the fitting uncertainty of up to 2 meV and the sensitivity of the calculated EQ peak position to temperature. Figure 4 also presents the calculated temperature-dependent linewidths alongside the experimentally determined FWHM, where each laser output power has been converted into temperature based on the corresponding spectral peak shift. We find that the experimental linewidth without laser illumination is offset to higher energy by 150 meV compared to the theoretical one. We attribute this to additional bulk absorption (*e.g.*, due to grain boundaries) and surface scattering losses (due to non-spherical shape) that are not considered in the simulations. Such losses may well be expected for the chemical synthesis process used to fabricate the Si NPs. Taking into account the offset, the shift in FWHM with increasing temperature shows a similar trend as that for the calculations based on the effect of temperature on the optical constants alone. This shows the consistency of the use of the thermally-induced refractive index changes as a probe of temperature in the spectral resonance analysis.

**Figure 4:**
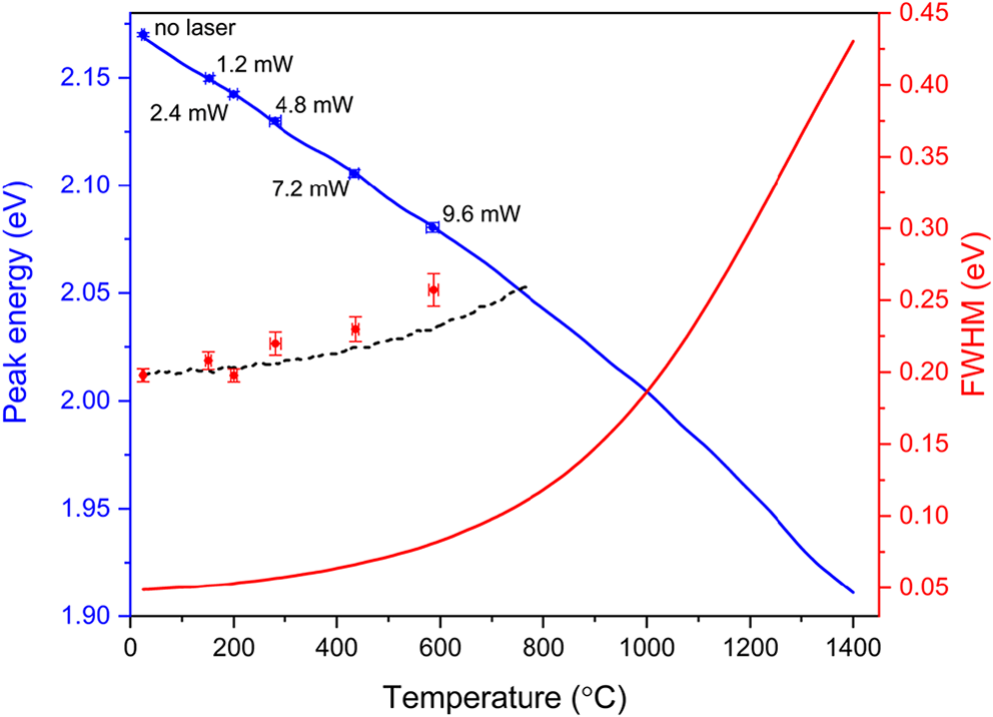
Peak energy of EQ resonant mode for Si NP (*d* = 250 ± 2 nm) *versus* temperature calculated using Mie model with temperature-dependent optical constants for Si (blue solid line). Experimental peak positions are overlaid on the graph (blue dots). Calculated FWHM linewidth of the EQ mode for temperatures between 25 and 1400 °C (red solid line). Experimental FWHM (red dots) plotted at temperatures derived from the peak energies (blue data). The black dashed line shows the calculated FWHM data offset to match the experimental data at 25 °C.

We then studied the reproducibility of the temperature-induced spectral shift and found three notable effects. First, in experiments on the same NP with laser powers up to 9.6 mW, the measured spectral shifts are generally reversible, and the morphology of the Si NP, as measured in the SEM, remained intact for laser exposure of at least 1 h (Figure S1). Second, performing experiments on different NPs with the same diameter on the same membrane yielded different results for the derived temperature increase as a function of laser power. And third, in some cases, we found that the particle had disappeared after illumination, leaving a hole in the membrane (Figure S2), suggesting it had melted and was ablated from the membrane.

To interpret these effects, we consider the fact that the heat flow rate away from Si NP strongly depends on the contact area of the spherical NP and the membrane. We postulate that the variations in measurements between different NPs are the result of variations in contact area. Indeed, variations in drop-casting, thereby induced drying forces and small variations in surface topography could result in nanoscale variations in contact area. To study the effect of contact area on the heat flow and steady-state temperature, we performed additional heat flow simulations. A 250-nm-diameter Si NP is placed on a 30 × 30 μm^2^ Si_3_N_4_ membrane with a thickness of 15 nm, and the contact area is varied from 1 to 700 nm^2^ (see the inset in Figure 5). A full description of the heat flow simulation as well as data for a larger contact area range can be found in the SI (Figures S11–S14).

**Figure 5:**
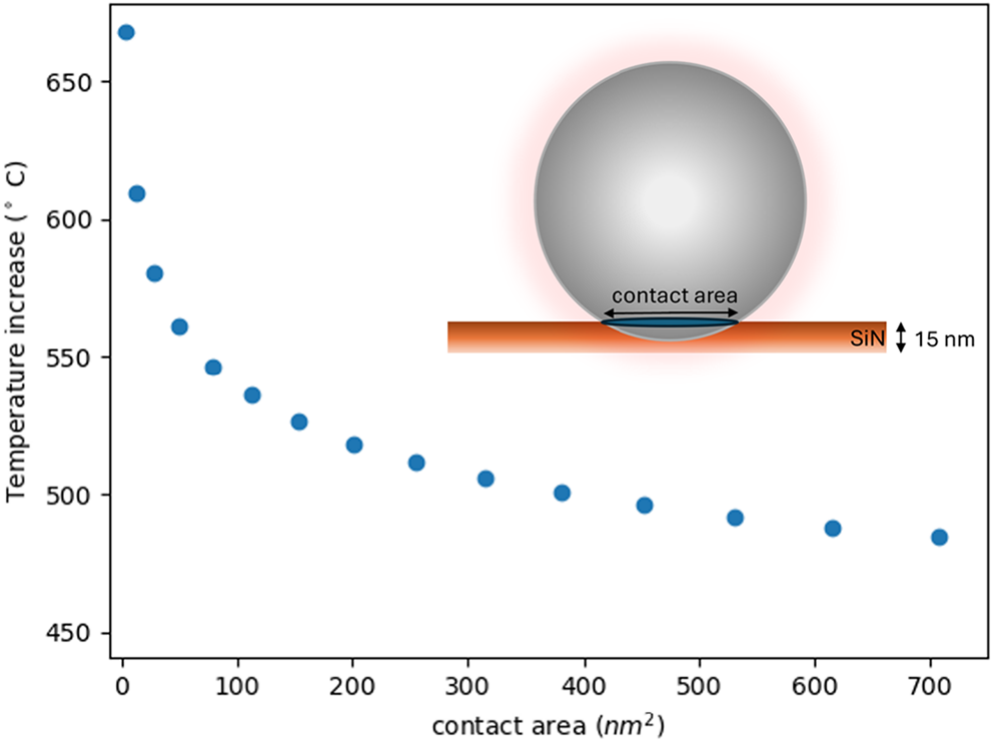
Steady-state temperature calculated from heat flow simulation for a Si NP NP (*d* = 250 nm) on a 15-nm-thin Si_3_N_4_ membrane for cw laser output power of 9.6 mW (λ = 442 nm) as a function of contact area between Si NP and Si_3_N_4_ membrane varying from 1 to 700 nm^2^. Inset: schematic of the geometry used in the simulations.

Figure 5 displays the steady-state temperature for a laser power of 9.6 mW as a function of contact area. For a very small contact area of 3 nm^2^, we find a temperature increase close to 670 °C; for an increasing contact area, the temperature rapidly decreases. Comparing experiment and simulation for laser powers in the range 4.8–9.6 mW, we estimate that for the Si NP in Figure 3, the contact area is about 100 nm^2^. Interestingly, for the two lowest output laser powers (1.2 and 2.4 mW), we find that the data is better represented by a much smaller point-like contact (3 nm^2^). Simulations using 4.8 mW laser power for a contact area smaller than 1 nm^2^ show that the Si NP rapidly heats up above the Si melting point (1412 °C). Indeed, the analytically calculated temperature increase of a Si NP in vacuum at a power density of 1 × 10^6^ W/cm^2^ is 350 K/ns, implying that the melting temperature of Si is reached within 39 ns.

Combining these results, we postulate that for the higher laser powers in this study, the silicon–membrane interface reshapes, creating a larger contact area, resulting in increased heat dissipation, which then reduces the steady-state temperature of the Si NP under illumination. The steady-state temperature that is achieved for a given laser power is then determined by a process in which the contact area rapidly reshapes in the beginning of the laser irradiation, as the NP rapidly heats up due to the small contact area, followed by a steady-state condition, in which heat input and flow are in balance, for a given contact area. The nanoscale reshaping effects may result from (heat-induced) plastic flow in the Si NP near the interface, the membrane, potentially due to surface atoms reorganization which can occur at substantially lower temperatures than required for bulk restructuring.^34^ Furthermore, (electron-induced) reshaping of the porous native oxide layer on the Si NP surface (Figure S15) could also result in reshaping of the Si-membrane contact area.^35–38^

## Conclusions

In this article, we introduce the use of SEM-CL in combination with laser excitation in the electron microscope. This allows for *in situ* investigation of photothermal effects with nanoscale spatial resolution, while concurrently probing optical properties through CL spectroscopy. We demonstrate that spatially and spectrally resolved CL measurements of resonant modes in Si nanospheres serve as sensitive probes of local temperature when irradiated with a 442 nm laser. A maximum temperature of up to 585 ± 12 °C is observed (9.6 mW laser output power, corresponding to ∼1 × 10^6^ W/cm^2^ at the substrate). The heat flow simulations compare well with the measured steady-state temperatures, assuming that the contact area between the Si NP and the membrane is increased from 3 nm^2^ for lower laser powers to 100 nm^2^ at higher laser powers. This implies that a complex reshaping process takes place in the initial phase of the laser irradiation, leading to an equilibrium geometry determined by the laser power. This model implies that small variations in the initial contact area between nanoparticle and membrane can have a strong effect on the final steady-state geometry and temperature under irradiation. This study highlights the value of combining experiment and simulation to probe heat flow at the nanoscale and paves the way for advanced *in situ* laser-CL studies in SEM to probe (photo)­thermal properties at the nanoscale.

## Supplementary Material



## Data Availability

The data that support the findings of this study are available from the corresponding author upon reasonable request.

## Notes

Additional SEM and TEM images of Si NPs, visualization of temperature-dependent refractive index of Si, optical simulations of substrate effect on Mie resonances in Si NP, laser-induced background subtraction for CL spectra under concurrent laser exposure, and additional heat flow simulations with COMSOL and methods (PDF).
