# Supplementary material for: Nanoscale Heat Flow and Thermometry in Laser-Heated Resonant Silicon Mie Nanospheres Probed with Spatially Resolved Cathodoluminescence Spectroscopy
Source: ACS Photonics. 2025 Sep 20;12(10):5668–74. doi: 10.1021/acsphotonics.5c01417 (PMC12532368; doi:10.1021/acsphotonics.5c01417)
Supplement: Supplementary file 1 [file ph5c01417_si_001.pdf]

Supporting Information

Nanoscale heat flow and thermometry in laser-heated resonant silicon Mie nanospheres probed with spatially-resolved cathodoluminescence spectroscopy

Saskia Fiedler[a,*], Loriane Monin[1], Hiroshi Sugimoto[2], Minoru Fujii[2], Wiebke Albrecht[1], and Albert Polman[1]

[1] Department of Sustainable Energy Materials, NWO-Institute AMOLF, Science Park 104, 1098 XG Amsterdam, the Netherlands

[2] Department of Electrical and Electronic Engineering, Graduate School of Engineering, Kobe University, Rokkodai, Nada, Kobe 657-8501, Japan

Table of Content

SEM images of laser-heated Si NP S2

Temperature-dependent refractive index of Si S2

Laser-induced background subtraction for CL spectra under concurrent laser exposure S3

Optical simulations of substrate effect on Mie resonances in Si NP S5

Additional COMSOL heat simulations S6

Optical simulations with MNPBEM17 as heat source input in COMSOL simulations S7

Heat flow simulations with COMSOL S7

TEM imaging of Si NP S10

References S11

SEM images of laser-heated Si NP

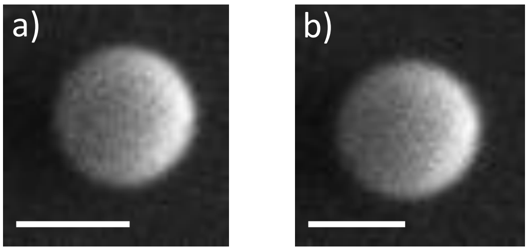

Figure S1. *SEM image of the Si NP (d = 250 nm) studied in figure 4 of the main text (a) before and (b) after laser exposure of several hours (λ = 442 nm, cw, laser output powers up to 9.6 mW), indicating no morphological changes took place. Scale bar denotes 200 nm.*

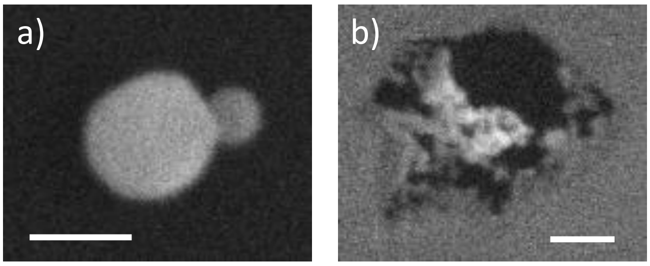

Figure S2. *Example of 250 nm-diameter Si NP (a) before and (b) after short 442 nm-laser illumination at 9.6 mW. Scale bar denotes 250 nm. NP was exposed to laser only for a few minutes.*

Temperature-dependent refractive index of Si

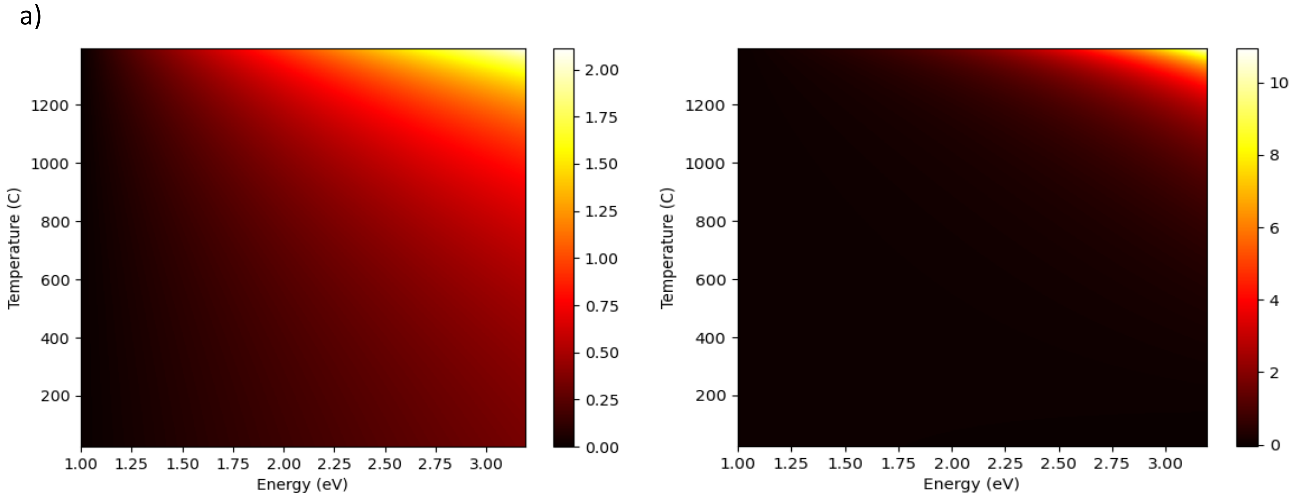

Figure S3. *Change of the (a) real and (b) imaginary part of the refractive index of silicon as a function of temperature and photon energy from Ref.* [1].

Laser-induced background subtraction for CL spectra under concurrent laser exposure

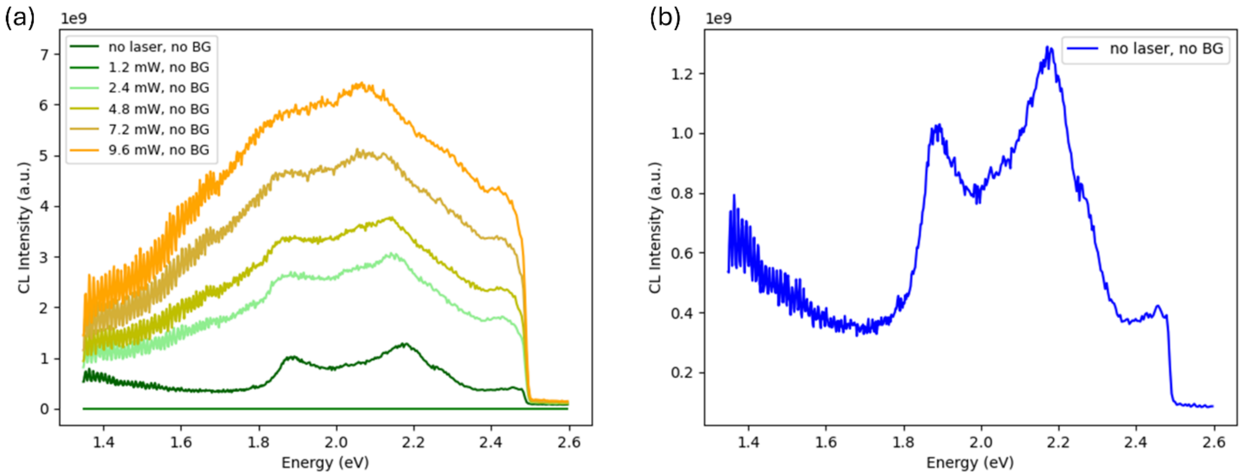

Figure S4. *(a) Raw CL spectra illustrating how increasing laser power enhances the background signal, masking the emission from a single NP. The sharp cut-off around 2.5 eV is due to a 500 nm long pass filter. (b) Magnified CL spectrum without laser excitation.*

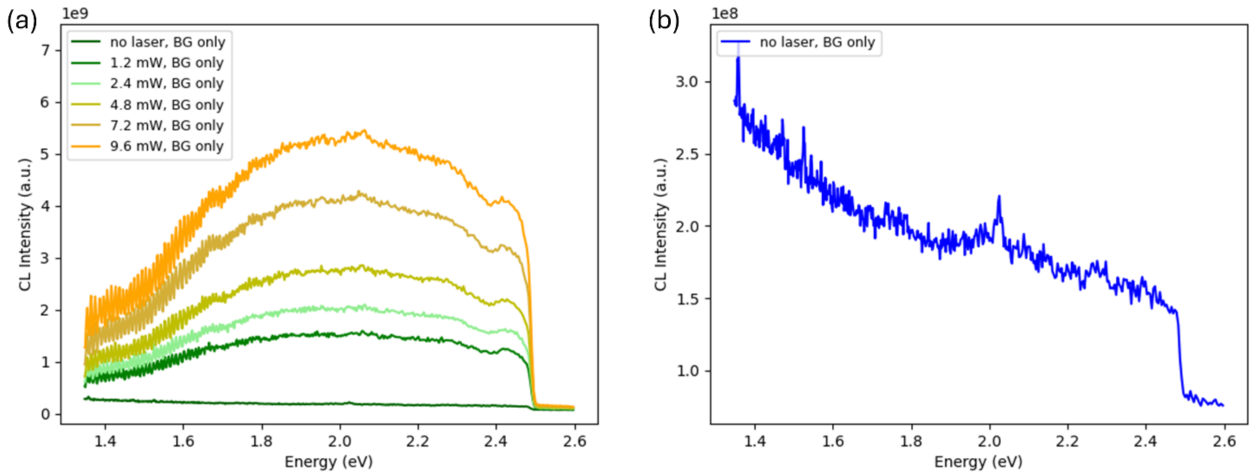

Figure S5. *(a) Laser-induced background. Sharp cut-off around 2.5 eV is due to a 500 nm long pass filter. (b) Magnified background without laser excitation.*

As shown in Figure S4, the raw CL signal from the Si NP becomes increasingly obscured by the laser-induced background as the laser power increases. To isolate the CL response, all spectra presented in Figure 3 have been background-corrected: the background signal was subtracted, and the resulting data were subsequently normalized to the EQ. The background spectra used for this correction, shown in Figure S5, were recorded at each laser power at the same sample position, but with the electron beam turned off. As the laser-induced background becomes more pronounced at higher laser powers, the signal-to-noise ratio of the background-corrected spectra decreases accordingly, leading to noisier CL spectra. To improve readability, the spectrum acquired under concurrent laser exposure at 9.6 mW

(Figure 3) was smoothed using a B-spline method. We note that the sharp cut-off around 2.5 eV is due to the 500 nm long pass filter in the collection path.

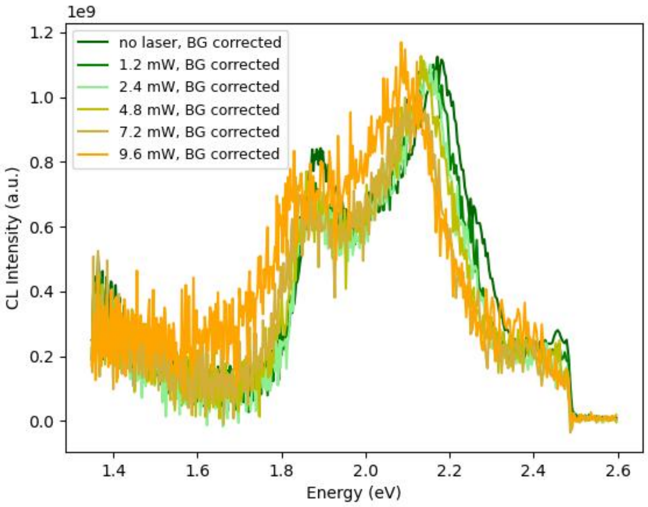

Figure S6. *Background-corrected CL spectra without normalization or smoothening.*

Figure S6 presents the background-corrected CL spectra, revealing that the CL intensity of the EQ mode remains largely unchanged with increasing laser power. In contrast, the MQ mode – partially overlapping with the broad ED – shows a decrease in intensity and increased spectral broadening. This behavior may be attributed to the combined influence of the overlapping EQ and ED modes. Additionally, the spectra were taken from the rim of the nanoparticle, where the EQ mode exhibits its maximum intensity, in contrast to the MQ mode, which is less dominant in that region.

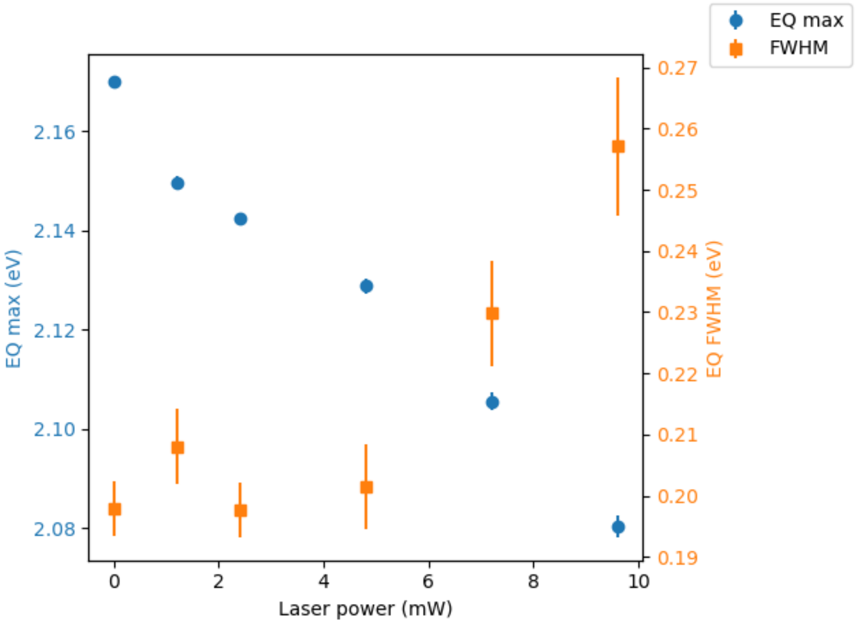

Figure S7. *EQ peak energy (blue) and linewidth (orange), extracted from the Lorentzian fit versus laser power.*

Optical simulations of substrate effect on Mie resonances in Si NP

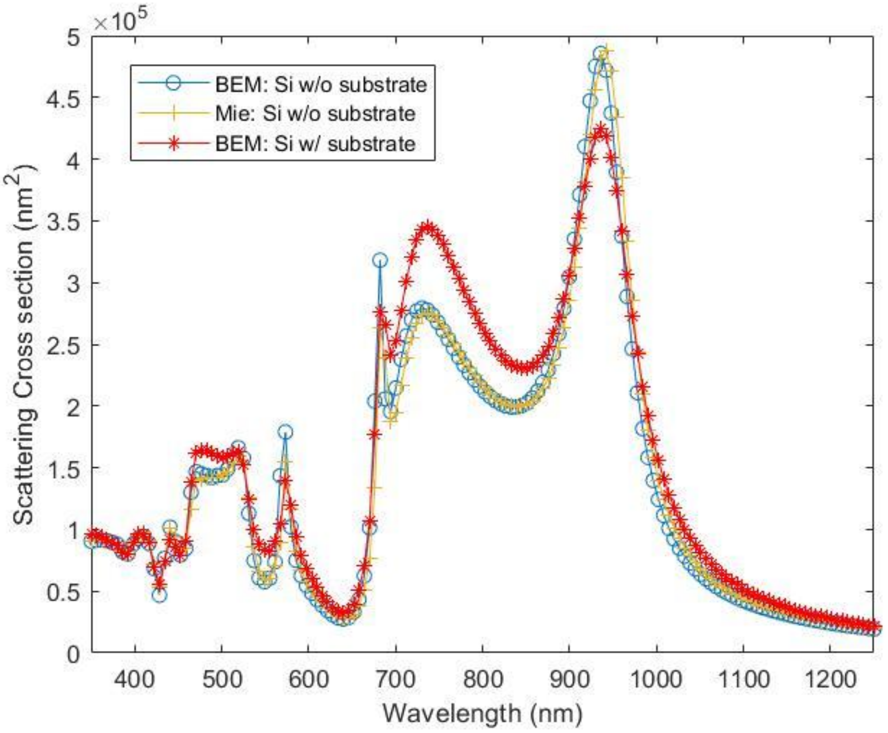

Figure S8. *Scattering cross-section of a Si NP (d = 250 ± 2 nm) in vacuum simulated with analytical Mie theory (yellow) and boundary element method (BEM, blue)[2], showing good agreement. Including a semi-infinite Si_3N_4 substrate in BEM (red) results in a slightly different intensity ratio between the Mie modes with minimal peak-broadening. Courtesy from P. Elli Stamatopoulou.*

Optical simulations show that the addition of a semi-infinite Si_3N_4 substrate only has a very small effect on the peak intensity and linewidths of the Mie resonances. We can therefore assume that for a 15 nm thin Si_3N4 membrane, the coupling to the substrate is relatively small.

Additional COMSOL heat simulations

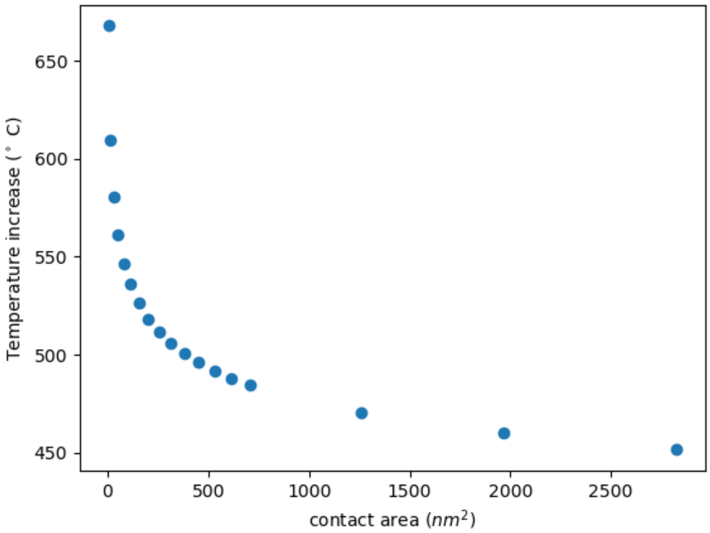

Figure S9. *COMSOL heat simulation of highest laser output power of 9.6 mW ranging to larger contact areas up to 2827 nm^2 (30 nm buried in the Si_3N_4 membrane) to show that a minimum temperature increase of 450°C is asymptotically reached.*

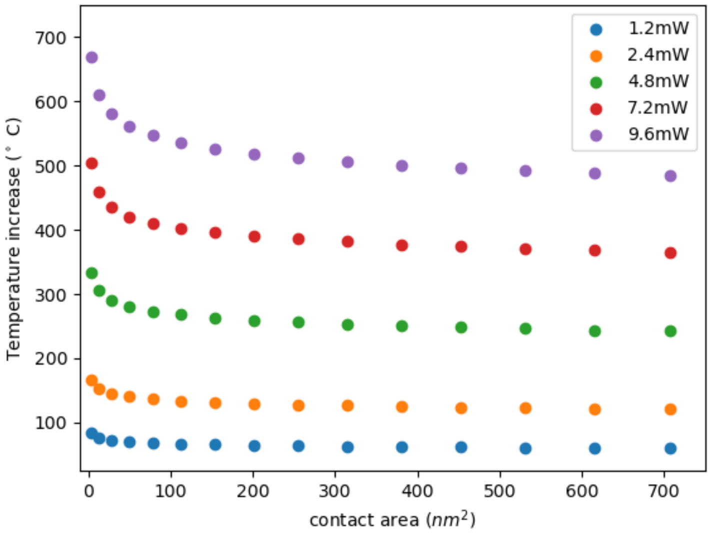

Figure S10. *COMSOL heat simulation. Simulated temperature increase as a function of contact area between the Si NP and the supporting 15 nm thin Si_3N_4 membrane for all experimentally used laser powers.*

Optical simulations with MNPBEM17 as heat source input in COMSOL simulations

The absorption and the scattering cross section of the Si NP were modelled with the Mie solver with retardation effect of the MATLAB toolbox MNPBEM17[3]. For the Si NP, the dielectric function of Green et al.[4] was used, and the surrounding medium was assigned a refractive index of 1 to represent vacuum. In this instance, because the nanosphere is very large, the substrate was neglected and we assume that the nanosphere is in a homogeneous environment, i.e. vacuum. The incoming light was a polarized along x. The resulting cross section calculations are shown in figure S11, which were used as a heat source input in the COMSOL simulations.

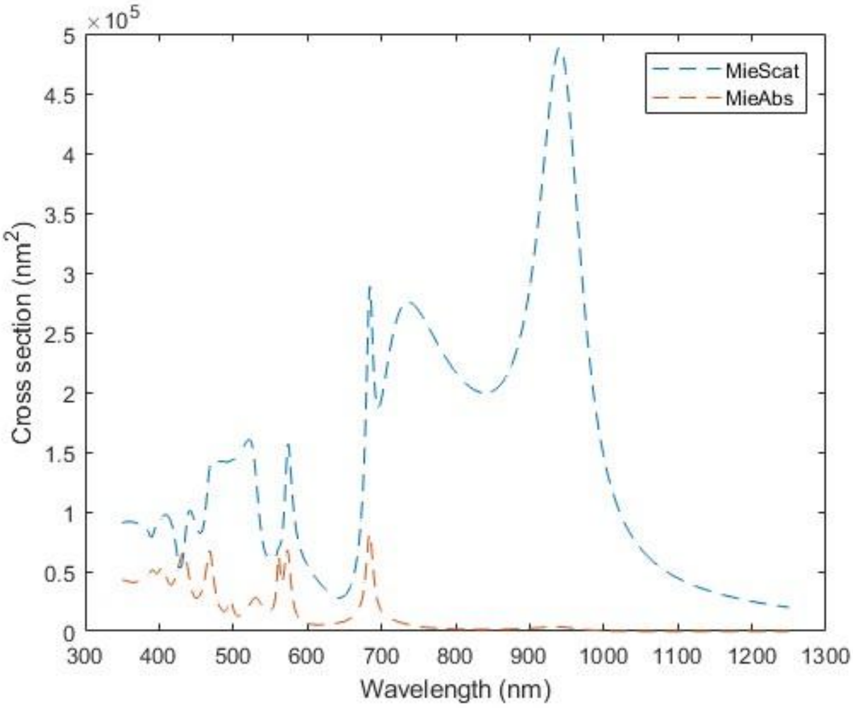

Figure S11. *Absorption and scattering cross section of a 250 nm-diameter Si NP in vacuum computed with MNPBEM17. The refractive index of the silicon was taken from Green et al.[4].*

Heat simulations with COMSOL

Physics

The Heat Transfer in Solids module from COMSOL v5.5 was used to solve the 3D heat diffusion equation at steady state. The heat source for the nanoparticle domain was defined as the product of the irradiance with the absorption cross section of the nanoparticle at 442 nm (Figure S11). The other material parameters are reported in Table S1.

Boundary conditions

The symmetry of the sphere was used to reduce computational cost such that only 1/4 of the sphere was simulated. A temperature boundary condition was applied to all the other boundaries which act as a heat sink. The temperature was fixed to 293 K to represent room temperature far from the silicon nanoparticle.

Meshing

To account for the large range of dimensions of the different simulation domains, a user defined free-tetrahedral mesh was built. First, the vacuum domains were meshed with a tetrahedral mesh with a normal size (minimum and maximum element size of 360 and 2000nm respectively). The 15 nm thick substrate was meshed with a minimum element size of 5 nm. The NP and the small domain around the NP were meshed with a minimum mesh size of 0.9 nm to ensure that the contact surface between the NP and the substrate is accurately meshed. Finally, a boundary mesh of eight layers was added to the boundary at which the heat flows from the nanoparticle to the substrate. The first layer was assigned a thickness of 0.5 nm and each subsequent layer thickness was increased by a factor 1.2. See figure S12 (a) for the domain mesh and figure S12 (b) for the faces assigned a boundary mesh.

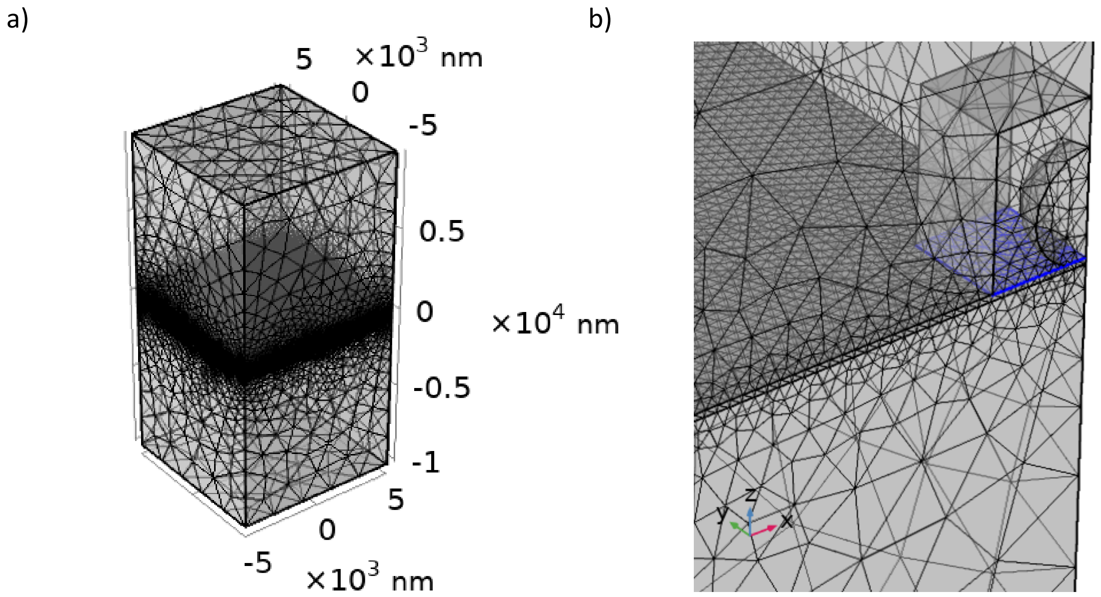

Figure S12*. Mesh of the complete domain (a) and (b) Zoom into the domain mesh of and around the nanosphere. The two boundaries in blue represent the boundaries on which the boundary layers were applied.*

Summary of the parameters used in the simulations

The thermal conductivity of silicon nitride highly depends on the film thickness and the deposition technique. In literature, values ranging from below 1 to 33 [W/(m*K)] have been reported[5]. However, most measurements are performed at room temperature or at low temperature (below 400 K)[6,7]. Simulations with free-standing silicon nitride thin films often use a thermal conductivity of 3-4 [W/(m*K)] at temperatures ranging from 300 K to 400 K. While no data could be found for silicon nitride thin films at high temperature, it is known that the thermal conductivity of amorphous thin films typically increases with temperature[6,8]. For example, Sikora et al. [8] reported that the thermal conductivity of a 100 nm Si_3N_4 membrane increases from 2 to 2.8 W/(m*K) when the temperature is increased from 100 K to 280 K[8]. In the present simulations we used the value of bulk silicon nitride of 32 W/(m*K) as it gave reasonable (and physically meaningful) temperature increases. For comparison, the temperature increase of Si_3N_4 with a thermal conductivity of 3.2 W/(m*K) as shown in figure S13 results in unrealistic temperature values.

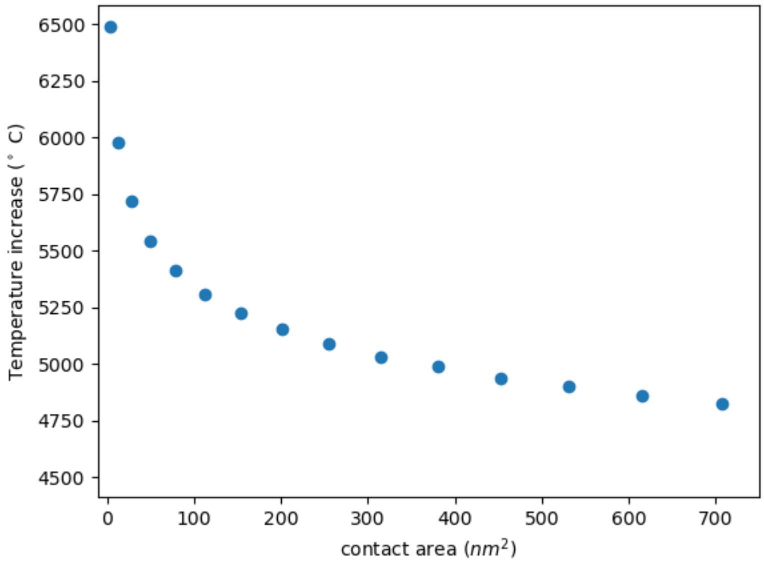

Figure S13. *Temperature increase of a 250 nm-sized silicon NP on a 15 nm Si_3N_4 membrane suspended in vacuum. The substrate was assigned a thermal conductivity of 3.2 [W/(m*K)].*

Table S1. *Input parameters for the heat simulations.*

Parameter	Value
Laser power[ǂ]	7.68, 5.76, 3.84, 1.92, 0.96 [mW]
Laser spot size radius	600 [nm]
Radius of the silicon sphere	125 [nm]
Substrate thickness	15 [nm]
Width and Length of the domain	10 [µm]
Absorption cross section at 442 nm	40010 [nm^2]
Thermal conductivity of silicon	148 [W/(m*K)]
Density of silicon	2.33 [g/cm^3]
Heat capacity of silicon	0.71 [J/(g*K)]
Thermal conductivity of silicon nitride	32 [W/(m*K)]
Density of silicon nitride	3.44 [g/cm^3] [‡]
Heat capacity of silicon nitride	0.7[J/(g*K)][9]

[ǂ] To account for losses in the optical path, the power input in the simulations was taken as 80% of the output power
[‡] As indicated by supplier

Investigating the influence of the domain size

Due to the finite domain size, it is important to ensure that this does not introduce artifacts in the computed solution. To check for this, the simulation for the highest power (output power of 9.6 mW, reduced by 20% to 7.68 mW) and the highest contact area (2825 nm^2) were computed with a domain size of 15 µm by 15 µm. Further, a heat sink was added to represent the silicon TEM grid frame (5 µm thickness). Figure S14 displays the results of this computation. As can be seen, the temperature profile does not extend further than 9 µm ensuring that the 10 µm simulation box used in all the simulations is sufficiently large enough to ensure full heat dissipation.

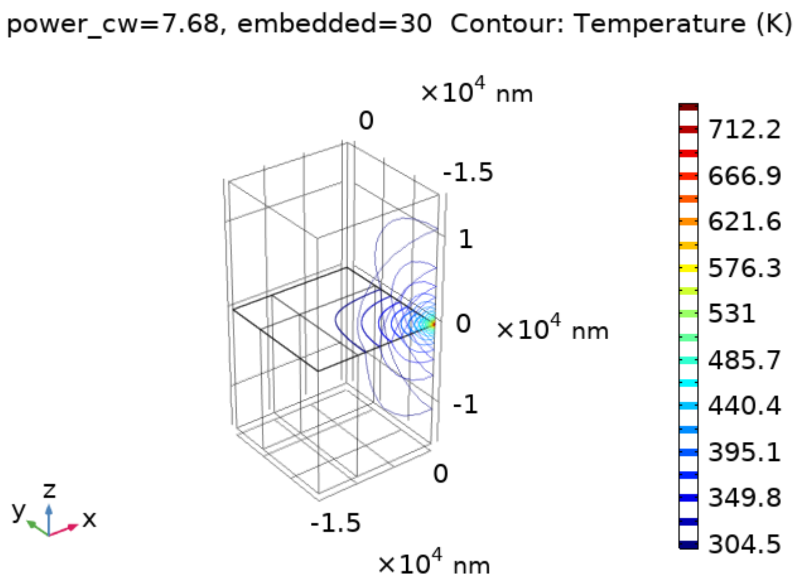

Figure S14. *Contour plot of the Temperature profile for a 15 µm by 15 µm showing that after 9 µm, the domain is at room temperature.*

TEM imaging of Si NP

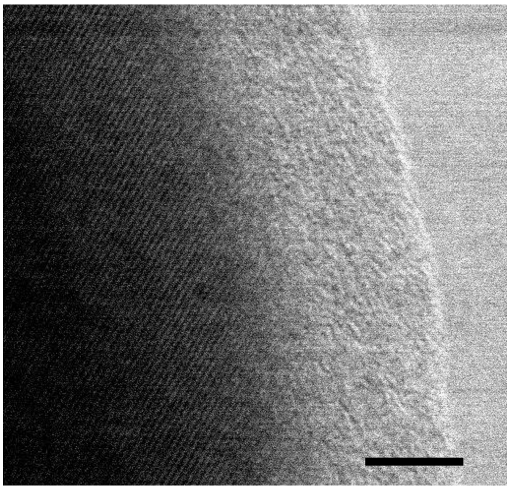

Figure S15. *Scanning TEM image (ABF) of the edge of a Si NP indicating high crystallinity Si surrounded by an approximately 5 nm thin SiO_2 shell. Scale bare denotes 5 nm.*

Scanning TEM image was collected in a probe corrected JOEL NeoARM using 200 keV and ~50 pA. ABF image was collected with 68-200 mrad.

References

(1) Jellison, G. E.; Modine, F. A. Optical Functions of Silicon at Elevated Temperatures. *Journal of Applied Physics* **1994**, *76* (6), 3758–3761.

(2) Hohenester, U. Nanophotonic Resonators in Stratified Media with the NANOBEM Toolbox. *Computer Physics Communications* **2023**, *293* (108949).

(3) Hohenester, U.; Trügler, A. MNPBEM – A Matlab toolbox for the simulation of plasmonic nanoparticles | Elsevier Enhanced Reader. *Computer Physics Communications* **2012**, 183, 370–381.

(4) Green, M. A. Self-Consistent Optical Parameters of Intrinsic Silicon at 300 K Including Temperature Coefficients. *Solar Energy Materials and Solar Cells* **2008**, *92* (11), 1305–1310.

(5) Reguer, A.; Bedu, F.; Nitsche, S.; Chaudanson, D.; Detailleur, B.; Dallaporta, H. Probing the Local Temperature by in Situ Electron Microscopy on a Heated Si3N4 Membrane. *Ultramicroscopy* **2009**, *110* (1), 61–66.

(6) Ftouni, H.; Blanc, C.; Tainoff, D.; Fefferman, A. D.; Defoort, M.; Lulla, K. J.; Richard, J.; Collin, E.; Bourgeois, O. Thermal Conductivity of Silicon Nitride Membranes Is Not Sensitive to Stress. *Phys. Rev. B* **2015**, *92* (12), 125439.

(7) Piller, M.; Sadeghi, P.; West, R. G.; Luhmann, N.; Martini, P.; Hansen, O.; Schmid, S. Thermal Radiation Dominated Heat Transfer in Nanomechanical Silicon Nitride Drum Resonators. *Applied Physics Letters* **2020**, *117* (3), 034101.

(8) Sikora, A.; Ftouni, H.; Richard, J.; Hébert, C.; Eon, D.; Omnès, F.; Bourgeois, O. Highly Sensitive Thermal Conductivity Measurements of Suspended Membranes (SiN and Diamond) Using a 3ω-Völklein Method. *Review of Scientific Instruments* **2012**, *83* (5), 054902.

(9) Hegedüs, N.; Balázsi, K.; Balázsi, C. Silicon Nitride and Hydrogenated Silicon Nitride Thin Films: A Review of Fabrication Methods and Applications. *Materials* **2021**, *14* (19), 5658.
